# Urban rooftops near sports pitches provide a safe haven for a declining shorebird

**DOI:** 10.1038/s41598-024-59693-1

**Published:** 2024-04-22

**Authors:** Franz Löffler, Jonas Brüggeshemke, Felix Maximilian Freienstein, Steffen Kämpfer, Thomas Fartmann

**Affiliations:** 1 Department of Biodiversity and Landscape Ecology, Osnabrück University, Barbarastraße 11, 49076 Osnabrück, Germany; 2 Institute of Biodiversity and Landscape Ecology (IBL), An der Kleimannbrücke 98, 48157 Münster, Germany

**Keywords:** Bird conservation, Eurasian Oystercatcher, Foraging habitat, Ground-nesting, *Haematopus ostralegus*, Predation risk, Ecology, Evolution, Ecology

## Abstract

Urbanisation has contributed to a severe decline in biodiversity worldwide. However, urban ecosystems can also play an important role in the conservation of threatened species, including ground-nesting birds such as the Eurasian Oystercatcher (*Haematopus ostralegus*). While the coastal populations of this shorebird have declined sharply, there is growing evidence that pairs nesting on urban flat roofs have high reproductive success. However, the reasons for rooftop nesting and the species’ habitat use in urban areas remain poorly understood. In this study, we investigate the territory selection and foraging behaviour of the Eurasian Oystercatcher in the city of Münster (NW Germany). All nesting sites were located on flat roofs (N = 24), most of which were covered with gravel. Overall, reproductive success was high. This was mainly because the roofs provided protection from mammalian predators, leading to increased nest and chick survival. Moreover, breeding performance in the study area was favoured by the proximity of sports pitches. According to our observations, they provided a large amount of easily accessible prey throughout the breeding season. Overall, our study highlights that the reproductive success of the Eurasian Oystercatcher in urban environments is highly dependent on both safe nesting sites on flat roofs and the availability of suitable foraging habitats. Although our study suggests that breeding in urban areas can be beneficial for the model organism, the species’ strong territory fidelity makes it very sensitive to the rapid environmental changes occurring in cities. The value of urban ecosystems for bird conservation should therefore be better integrated into urban planning and management.

## Introduction

With increasing human population and rapid economic growth, urban areas have expanded during the last decades all over the globe^1,2^. Since urban sprawl has led to a severe loss of natural and semi-natural habitats, urbanisation is considered to be one of the most serious threats to biodiversity^3,4^. Although cities are usually considered hostile environments for most taxa and exhibit a high level of human disturbance, urbanisation can also contribute to the emergence of novel ecosystems that can play an important role in maintaining biodiversity, including the conservation of threatened species (e.g.^5–7^). This is especially true when cities are surrounded by intensively used landscapes where suitable habitats are limited for most species^8,9^. Moreover, recent studies found positive effects of reduced anthropogenic activity during the COVID-19 lockdown on urban wildlife (e.g.^10,11^). For instance, it has been shown that shorebirds benefitted from reduced human disturbance in urban beach ecosystems^11,12^. By contrast, the study of Seress et al.^13^ revealed that the breeding success of an urban adapter species did not change when human disturbance decreased during the lockdown.

Birds are an important component of urban biodiversity and are among the best-studied animal groups in urban ecosystems (e.g.^14,15^). Despite the great challenges of living in cities, many bird species have successfully adapted to urban habitats^16–18^. In particular, cities can provide suitable nesting conditions for habitat generalists and a high abundance of shrub and cavity-breeding birds (e.g.^8,19–21^). However, it has also been reported that some ground-nesting birds, which have suffered severe declines in European agricultural landscapes^22^, can breed successfully in urban areas (e.g.^23,24^).

This is also the case for the Eurasian Oystercatcher (*Haematopus ostralegus*, hereinafter referred to as Oystercatcher), which was originally restricted to coastal areas but has shifted inland along the floodplains of major rivers in Central and Western Europe during the last decades (e.g.^25–27^). While the coastal Oystercatcher population is declining at an alarming rate, the inland breeding population is increasing throughout Europe (e.g.^28–31^). According to Gedeon et al.^26^, inland breeding Oystercatchers account for at least 10% of the German breeding population. Several authors have observed that inland Oystercatcher populations often use urban flat roofs for breeding (e.g.^32–35^). Rooftop nesting is relatively common among seabirds such as gulls and terns (e.g.^36–38^). However, it rarely occurs among waders^39^. The Oystercatcher is an exception as it is one of the few European waders that feeds its chicks, which therefore do not need direct access to foraging habitats during the rearing period^40^. Although the Oystercatcher is among the best-studied shorebirds in Europe^31,41^, the breeding ecology of Oystercatchers nesting in urban environments is still poorly understood.

In this study, we investigated territory selection and foraging behaviour of the Oystercatcher in the city of Münster (NW Germany) in 2020. We compared the habitat use of both successful and unsuccessful breeding pairs to the habitat conditions in random sites. In particular, we aimed to identify the environmental factors that determine nesting success and improve our understanding of the species’ foraging preferences in urban areas. As we expected that the risk of predation is significantly reduced when Oystercatchers breed on rooftops (cf.^37,38^), we hypothesised that this strategy could result in higher reproductive success of the species. Furthermore, we assumed that a large proportion of urban green space is essential to provide sufficient food throughout the breeding season (cf.^33,34^). In line with these hypotheses, we assert that cities can provide good breeding conditions for the Oystercatcher but also address potential limitations of our study. The results of our study can be used to draw conclusions about measures that can be taken to mitigate the negative population trend of the species.

## Background information

### Study species

The Eurasian Oystercatcher (*Haematopus ostralegus*) is a migratory shorebird over most of its breeding range that includes much of the Palearctic. The European breeding population of the nominate subspecies *Haematopus ostralegus ostralegus* is currently estimated to be at least 300,000 breeding pairs, with a clear stronghold in the UK and Norway as well as the Wadden Sea in Germany and the Netherlands^27,31, 42^. The inland shift of the species has often been attributed to a population increase that took place until the 1980s, but is likely also due to reduced reproductive success following severe environmental changes in coastal habitats (e.g.^25,26,31,43^^,44^). Oystercatchers are long-lived birds that have a strong territory fidelity. Breeding mainly takes place in sparsely vegetated coastal habitats, such as salt marshes, beaches and dunes^40,45^. In addition to habitats associated with freshwater marshes, flat gravel rooftops are among the main breeding habitats in inland areas, especially in urban environments^27,29,34,45^. The availability of foraging habitats with high prey abundance plays an important role for successful breeding of the Oystercatcher^31,46^. On the coast, where the species’ diet consists mainly of marine molluscs and worms, foraging is highly dependent on tidal dynamics^40,45^. By contrast, inland breeders feed primarily on earthworms captured from short-turf grassland and urban green infrastructure such as lawns and sports pitches^40,43,45,47^. As a result of negative population trends throughout most of its range, the Oystercatcher is now listed as vulnerable in Europe and near threatened globally^31,42^.

### Study area

**Figure 1:**
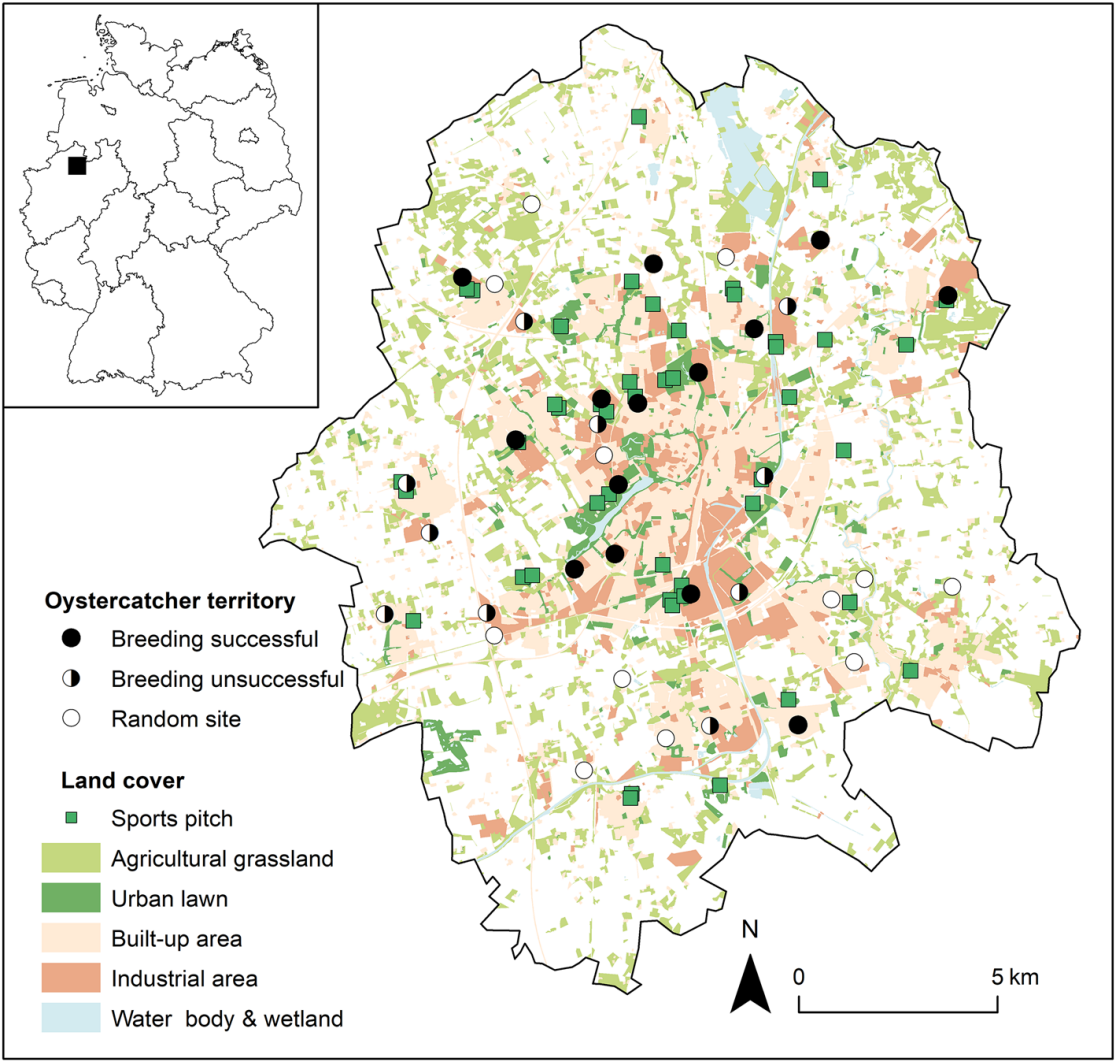
Location of Oystercatcher territories and random sites and the prevailing land-cover types in the city of Münster (NW Germany), for more details see Table 1.

The study was conducted in the city of Münster (North Rhine-Westphalia, Germany, 51° 58ʹʹ N, 7° 38ʹʹ E; 39–99 m a.s.l, Fig. 1). Münster is a medium-sized, rapidly growing city with a human population of 320,000 inhabitants in an area of 303 km^2^. It is located in the North German Plain, 200 km south of the Wadden Sea coast. The study area covers the whole municipal area of the city of Münster (~ 300 km^2^). The climate in the study area is suboceanic with a mean annual temperature of 10.4 °C and an average annual precipitation of 763 mm (1991–2020, weather station Münster/Osnabrück^48^). Since the mid-twentieth century, the study area has been severely affected by agricultural intensification and urban sprawl^28^. Today, it is largely covered by built-up areas (34%) surrounded by intensive arable land (46%) and forests (18%), water bodies and wetlands together cover only 2%. Among the latter, the EU Bird Sanctuary’ Rieselfelder Münster’ is one of the most important inland stopover areas for shorebirds in north-western Germany. The first evidence of breeding of the Oystercatcher in the study area dates back to 1969^49^. Since then, the local Oystercatcher population has steadily increased^28^. Some breeding pairs in the study areas nested in the same location for at least 20 years (own observation).

## Materials and methods

### Bird census

Oystercatcher territories were located using standardised territory mapping. Bird census was done under favourable weather conditions between 6 and 10 a.m. The whole study area was searched for Oystercatchers over four surveys carried out between early April and mid-June 2020 with at least 7 days between two consecutive surveys^50,51^. Following Bibby et al.^50^, all observations of territorial behaviour (e.g. breeding or feeding adults, alarming birds and pairs at potential nesting sites) were recorded with their location during each survey. In order to separate the territories of different birds in close proximity, particular attention was paid to simultaneous observations of territorial behaviour^52^. If birds showing territorial behaviour were detected at potential nesting sites during at least two of the four surveys, breeding was assumed^51^.

To assess nesting success, territories were additionally checked weekly between mid-June and mid-July for the presence of juvenile birds^30^. Nesting success was confirmed if at least one fledgling was detected in a territory during this period^53^. If adults left the nesting site prior to the fledging of the chicks, breeding was considered unsuccessful. In the case of successful nesting, we also counted the number of fledglings in order to calculate the reproductive output of the Oystercatcher population in the study area.

In further analyses, we compared the habitat characteristics within territories of successful (N = 14) and unsuccessful breeding pairs (N = 10) and random sites (N = 12). Following the procedure of previous studies (e.g.^37,54^), the random sites were selected from all available flat roofs in the study area using the ‘create random points’ tool in ArcGIS 10.8.1. They thus represent a random selection of potential nesting sites in the city of Münster, which were not occupied by Oystercatchers, and reflect the general environmental conditions of the entire study area.

### Foraging preferences

During the main rearing period (early June to mid-July^40,45^), we studied the foraging preferences of the Oystercatcher in a total of twelve territories. We examined foraging in the nearest (i) urban lawn, (ii) agricultural grassland and (iii) sports pitch (playing surface with natural grass turf, in the study area mainly used for playing football) in the vicinity of the nesting site for 15 min in each of the three habitat types. These habitats are among the most important foraging habitats for the Oystercatcher in urban areas (e.g.^33,34,41,47^). During the observation period, we measured the duration of foraging activity (time in minutes) and counted the foraging success (catches/attempt) of the Oystercatcher.

### Environmental parameters

The environmental conditions within Oystercatcher territories and random sites were determined using digital landscape models (DLM/ATKIS, scale: 1:10,000)^55^. For this purpose, the proportions of the prevailing terrestrial land-cover types were calculated within a 1 km radius around the centre of each breeding territory (i.e. nesting site) and random site. The radius corresponds to the large territories of the Oystercatcher used for foraging^40^. The following seven land-cover types were distinguished: (i) arable land, (ii) agricultural grassland, (iii) forest and shrub, (iv) built-up area, (v) urban lawn, (vi) industrial area, (vii) water body and marshland. These data were further used to calculate the Shannon index (Hʹ) as a measure of landscape diversity^56^:

$${H}^{\prime}= -\sum_{i}{p}_{i}{\text{ln}}{p}_{i} ,$$

with *p*_*i*_ = *n*_*i*_*/N* where *N* is the overall area of the 1 km buffer and *n*_*i*_ is the area of the respective land-cover types in the buffer area.

We also recorded the size, elevation above ground and substrate type of all rooftops occupied by Oystercatchers as well as the flat roofs selected as random sites. In addition, we measured the distance to the nearest available sports pitch for both occupied nesting sites and random sites (i.e. potential nesting sites). Only pitches with natural grass turf were considered. We calculated the Normalised Difference Vegetation Index (NDVI) based on Sentinel-2 satellite imageries to quantify the effect of drought on potential foraging habitats during the breeding season (cf.^57^). Mean NDVI was calculated for the area of (i) agricultural grasslands and (ii) urban lawns within the 1 km buffer and (iii) the nearest available sports pitch. These calculations were done using the zonal statistics tool in ArcGIS 10.8.1 for early May, June and late July, respectively.

### Statistical analyses

Statistical analyses were performed using R 4.1.2^58^. If the data were normally distributed, statistical differences in environmental parameters between successful and unsuccessful breeding pairs of the Oystercatcher and random sites were tested using ANOVA with Tukey’s test as a post-hoc test. Otherwise, we used the Kruskal–Wallis H test with Dunn’s test as a post-hoc test. We used repeated measures ANOVA on ranks followed by the Holm-Sidak test as a post-hoc test to identify differences in the NDVI of potential foraging habitats in Oystercatcher territories. Differences in foraging activity and success in the habitats that were in fact visited to capture prey were tested using the Wilcoxon Signed Rank Test and the Mann–Whitney U test, respectively.

We used multivariable generalised linear models (GLMs) (binomial error structure) to identify environmental factors that explain the occurrence of successful and unsuccessful breeding pairs against random sites, respectively. To identify the most important predictors, all possible combinations of the sampled environmental parameters were tested, resulting in a number of different candidate models. These were ranked based on Akaike’s information criterion for small sample sizes (AICc) using the ‘dredge’ function (R package MuMIn)^59^. Only top-ranked models with ΔAICc < 2 were used for model averaging^60^. All predictor variables were scaled prior to analyses to ensure comparability of model coefficients of different parameters. To avoid multicollinearity in the models, Spearman’s rank correlations (r_s_) were applied prior to the GLM analyses. Combinations of highly intercorrelated variables (|r_s_| > 0.5) were excluded from the modelling process. In addition, the number of predictors in the different candidate models was limited to three parameters to avoid overfitting.

## Results

### Breeding habitats

A total of 24 breeding Oystercatcher pairs were recorded in the study area. In 58% of the pairs (N = 14), at least one young successfully fledged; in the remaining pairs (N = 10), breeding failed. Except for one pair with two chicks, breeding pairs reared one chick, resulting in an overall reproductive success of 0.6 fledglings per breeding pair. All Oystercatcher nesting sites were located on flat roofs, the majority of which (88%) were covered with gravel. Breeding was unsuccessful in two of the three pairs nesting on non-gravelled roofs. Whereas no differences were found in the height and size of the occupied rooftops, clear differences were observed in the landscape surrounding the roofs used for nesting (Table 1). The territories of successful breeding pairs were characterised by a larger proportion of industrial areas (mean ± SE: 10.0 ± 2.1%) and urban lawns (mean ± SE: 19.5 ± 5.3%) compared to the random sites (mean ± SE: 3.4 ± 1.3% and 9.8 ± 3.1%, respectively). Their nesting sites were also closer to the nearest sports pitch (mean ± SE: 445 ± 126 m) than potential nesting sites on the randomly selected rooftops ((mean ± SE: 1230 ± 188 m).

**Table 1:** Mean (± standard error [SE]) of the environmental parameters within the three study groups: breeding successful (N = 14), breeding unsuccessful (N = 10), random site (N = 12).

Parameter	Mean ± SE			p
	Breeding successful	Breeding unsuccessful	Random site	
Land cover [%]				
Built-up area	31.6 ± 4.0	30.1 ± 4.5	22.7 ± 4.4	n.s.^1^
**Industrial area**	**19.5 ± 5.3**^**a**^	**18.5 ± 2.9**^**ab**^	**9.8 ± 3.1**^**b**^	*^2^
**Urban lawn**	**10.0 ± 2.1**^**a**^	**6.1 ± 1.5**^**ab**^	**3.4 ± 1.3**^**b**^	**^2^
Agricultural grassland	10.7 ± 2.2	8.6 ± 1.8	16.4 ± 2.6	n.s.^1^
Arable land	18.0 ± 4.6	22.2 ± 5.2	31.5 ± 5.5	n.s.^1^
Forest/shrub	9.0 ± 2.1	12.1 ± 2.8	15.1 ± 2.6	n.s.^1^
Water body/marshland	2.1 ± 0.8	1.4 ± 0.5	1.1 ± 0.3	n.s.^1^
Landscape diversity [Hʹ]	1.5 ± 0.0	1.5 ± 0.1	1.5 ± 0.04	n.s.^2^
Roof size [m^2^]	961 ± 218	2550 ± 1229	1257 ± 695	n.s.^1^
Roof height [m]	8.4 ± 1.2	9.1 ± 1. 2	7.5 ± 1.5	n.s.^1^
**Distance sports pitch [m]**	**445 ± 126**^**a**^	**715 ± 154**^**ab**^	**1230 ± 188**^**b**^	**^1^

Land cover [%] was measured within a 1 km radius around nesting sites and random sites, respectively.

Differences among the groups were tested using ANOVA (post-hoc test Tukey’s test^1^) or the Kruskal–Wallis *H* test^2^ (post-hoc test Dunn’s test).

Different letters indicate significant differences among the study groups (p < 0.05 highlighted by bold type). *n.s.* not significant, *p < 0.05, **p < 0.01.

**Figure 2:**
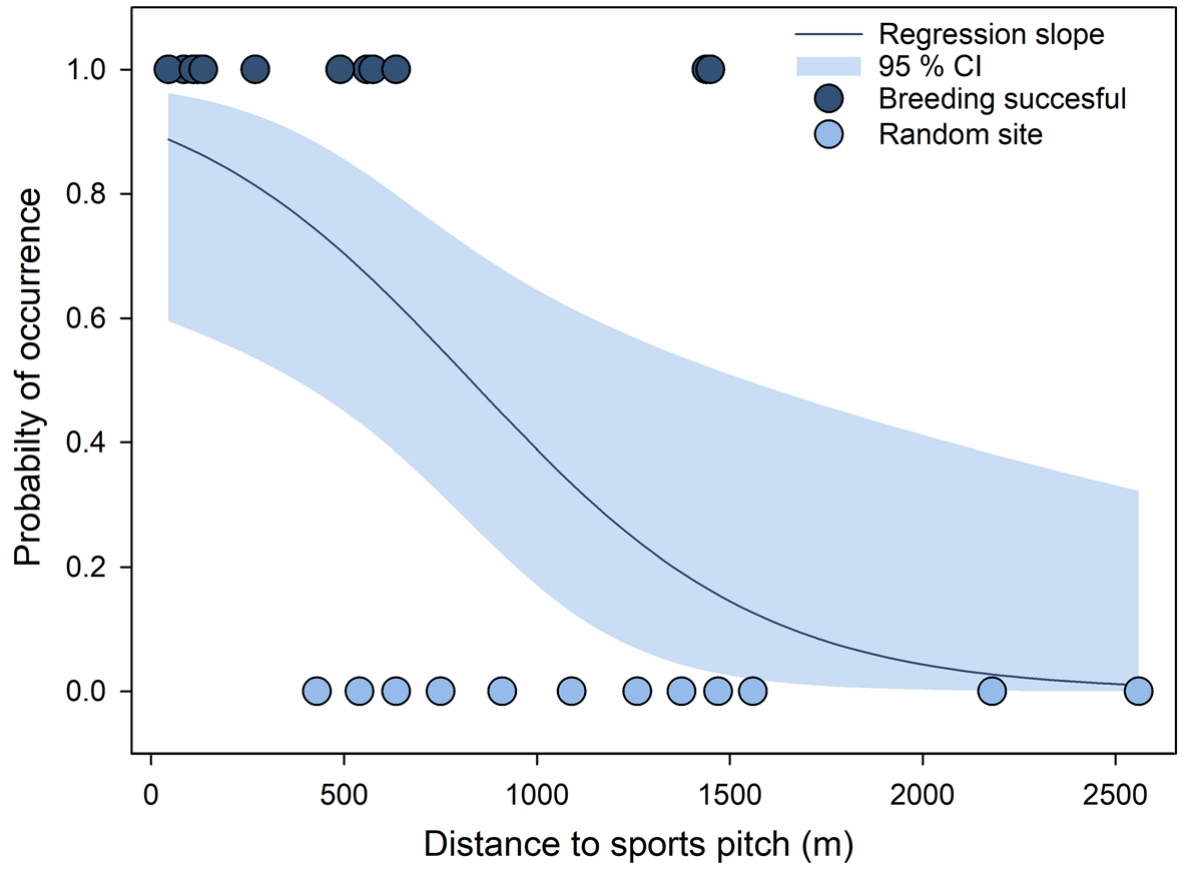
Relationship between the occurrence of successful breeding pairs and the distance to the nearest sports pitch based on generalised linear model with binomial error structure (p < 0.05). Successful breeding pairs (N = 14, dark blue) vs. random sites (N = 12, light blue) formed the two categories of the response variable, Regression slope (± 95% confidence interval = CI) fitted based on the GLM output y = 1/(1 + exp − (− 1.144 − 7.49 × distance to sports pitch)), for more details see Table 2a.

Based on the GLM analyses, the distance between the nesting site and the nearest sports pitch was identified as the key factor for successful breeding (Fig. 2, Table 2). By contrast, we did not detect any environmental factors explaining unsuccessful breeding.

**Table 2:** Results of the GLM analyses (binomial error structure) investigating the relationship between the occurrence of (a) territories with nesting success (N = 14) or (b) territories without nesting success (N = 10) and random sites (N = 12).

Parameter	Estimate	SE	Z	p
(a) Breeding successful vs. random sites				
(Intercept)	0.04	0.56	0.08	n.s.
Distance sports pitch	− 2.53	1.05	− 2.41	*
Agricultural grassland cover	− 1.46	0.77	− 1.90	n.s.
Pseudo R^2^ (McFadden) = 0.43				
(b) Breeding unsuccessful vs. random sites				
(Intercept)	− 0.64	0.76	0.85	n.s.
Agricultural grassland cover	− 2.03	1.16	− 1.75	n.s.
Distance sports pitch	− 1.65	1.07	− 1.55	n.s.
Roof size	0.78	0.64	1.21	n.s.
Landscape diversity	0.94	0.95	0.98	n.s.
Pseudo R^2^ (McFadden) = 0.35–0.42				

Model averaged coefficients (conditional average) derived from the top-ranked models (ΔAICc < 2) are shown.

Statistical significances are indicated as follows: *n.s.* not significant, *p < 0.05.

### Foraging habitats

**Figure 3:**
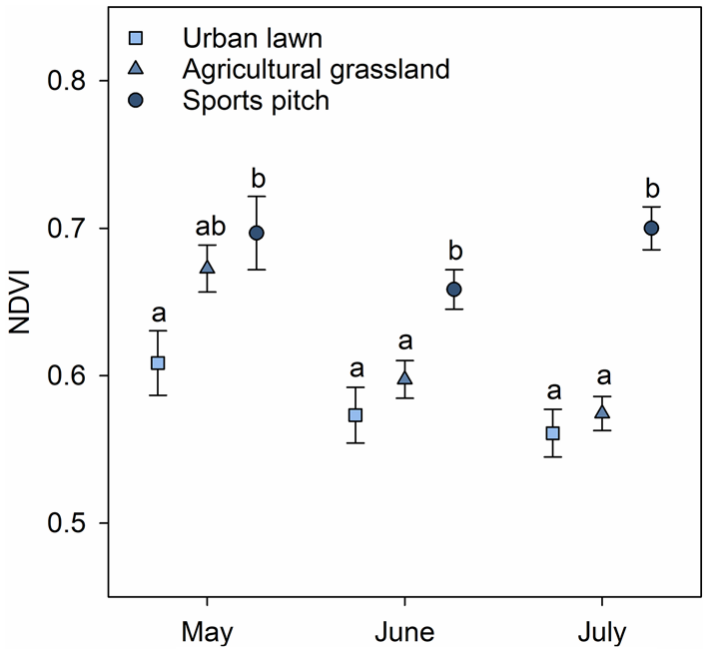
Differences in the Normalized Difference Vegetation Index (NDVI) between potential foraging habitats of the Oystercatcher in the study area: (i) urban lawns (light blue square) and (ii) agricultural grasslands (medium blue triangle) within a 1 km radius around the centre of each breeding territory and (iii) the nearest available sports pitch (dark blue point) during the breeding season of the species in the study area (May to July). Mean ± SE are shown. Different letters indicate statistical significances (p < 0.05) between the groups.

**Figure 4:**
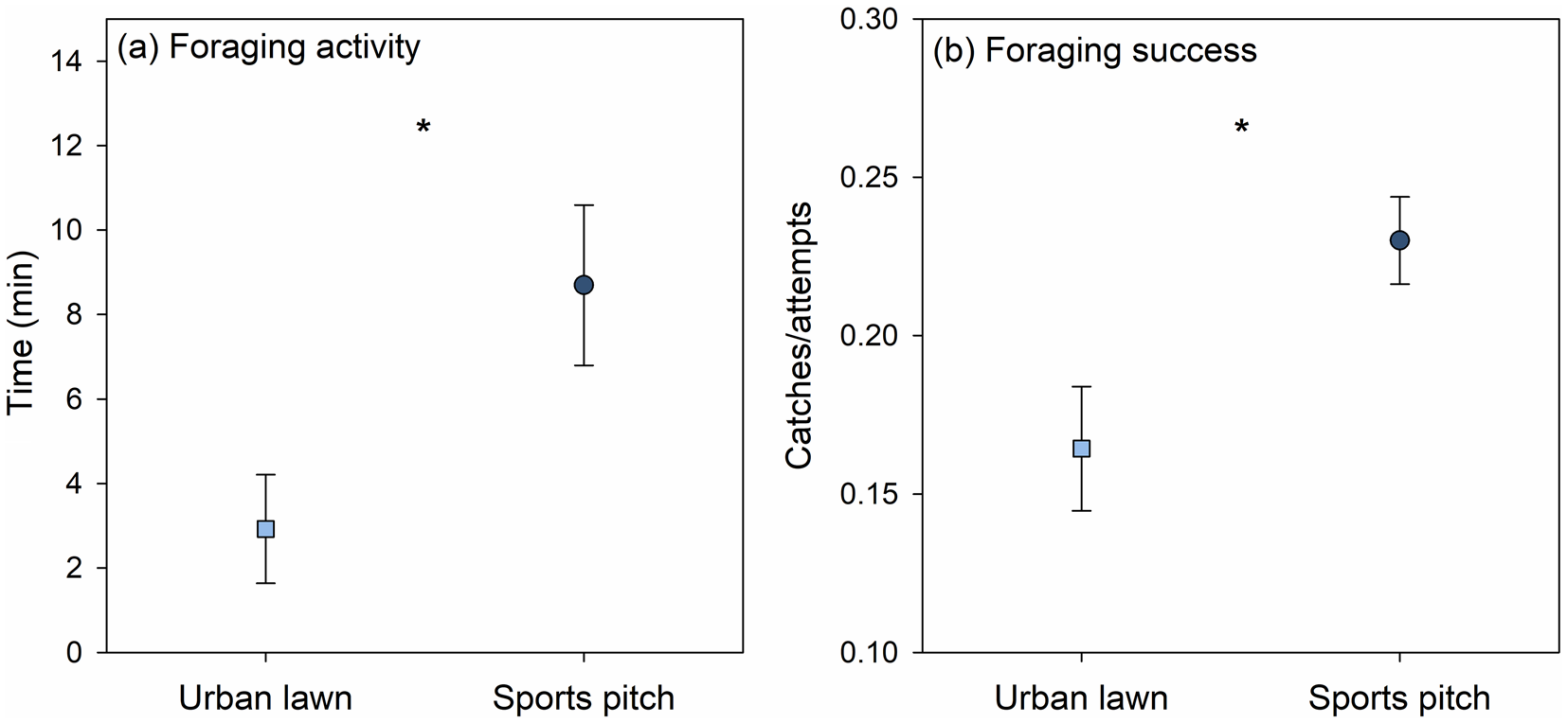
Differences in foraging activity and success of the Oystercatcher between urban lawns (light blue square) and sports pitches (dark blue point) during the rearing period of the species in the study area (June to July). Mean ± SE are shown. Statistical significances between the groups are indicated by asterisks (*p < 0.05).

Among the potential foraging habitats, the NDVI of urban lawns and agricultural grasslands clearly decreased from May to July (Fig. 3). By contrast, the NDVI of sports pitches did not change during this period. Consequently, pitches were characterised by higher NDVI values than urban lawns throughout the whole breeding season. In June and July, they also had higher NDVI values compared to agricultural grasslands. Both foraging activity and foraging success were also higher on sports pitches than on urban lawns during the rearing period (Fig. 4). No foraging activity was observed on agricultural grasslands during this period.

## Discussion

Compared to the very low reproductive success of the Oystercatcher in its coastal habitats in recent years^30,43,45^, we detected high nesting success of Oystercatchers nesting on urban rooftops in our study. Breeding mainly took place on flat gravel roofs situated in close proximity to foraging habitats. Successful breeding was particularly favoured by a short distance to sports pitches. By contrast, our study did not detect environmental factors that explain unsuccessful breeding in urban areas.

Roof nesting of Eurasian Oystercatchers has been reported from several regions within their range and also occurs in other Oystercatcher species around the world (e.g.^26,32,33,41^). In general, Oystercatchers are very flexible in their choice of nesting sites^40,41,45^. As gravel roofs have a similar structure compared to natural breeding habitats such as shingle beaches, it is not surprising that they were used for nesting in our study. However, confirming the study of Dijkstra and Dillerop^47^, we observed that non-gravelled roofs were also sometimes used for nesting. Although Oystercatcher chicks sometimes can die by falling or jumping from the roofs (e.g.^47^), our study revealed that the reproductive success of roof-nesting Oystercatchers (0.6 fledglings/pair was far above the average of the reproductive outcome in coastal (~ 0.18 fledglings/pair on islands and ~ 0.11 fledglings/pair on the mainland coast)^30^, and agricultural habitats (~ 0.11 fledglings/pair)^30,32^. This is consistent with other authors that highlighted the high reproductive success of Oystercatcher populations in urban areas (e.g.^33,34,43^).

The main advantage of rooftop nesting is that it provides effective protection from mammalian predators such as the Red Fox (*Vulpes vulpes*), whose populations have strongly increased in recent decades, contributing to the dramatic decline of many ground-nesting birds (e.g.^61–63^). It is well known that breeding in habitats that cannot be reached by predatory mammals is an effective breeding strategy for many ground-nesting birds^38,44,64^. This is also true for most shorebirds, such as the Oystercatcher, which currently has very low breeding success throughout most of its range except for some islands that are largely devoid of predatory mammals^24,30,65,66^. By contrast, several studies have found that the nesting success of ground-nesting birds can be higher in urban habitats due to lower abundance of mammalian predators (e.g.^23,67,68^). This is especially true for Oystercatchers nesting on rooftops, which are completely inaccessible to predatory mammals and therefore provide effective protection for juvenile birds, provided that they do not leave the rooftop until they fledge (Ref.^47^, cf.^38^). Moreover, human disturbance is also known to be detrimental to the breeding performance of the Oystercatcher (e.g.^31,40^). This especially applies to habitats with high human activity. While it has recently been found that the abundance of shorebirds in urban beach ecosystems increased during the COVID-19 pandemic due to a reduced frequency of people^12^, roof-nesting Oystercatchers are protected from human disturbance at any time, because there usually is no public access to rooftops.

Nevertheless, eggs and chicks of roof-nesting Oystercatchers can still be preyed upon by avian predators such as corvids or gulls^40,43, 45^. Although we did not detect any environmental factors that contributed to breeding losses, predation by corvids, which are usually abundant in urban environments and were very common in the study area, was probably one of the reasons for failed breeding in our study (cf.^68^). In general, the impact of avian predation on the nest success and chick survival of ground-nesting birds is expected to be less severe, particularly for Oystercatchers, which exhibit aggressive defence towards predators (e.g.^40,69,70^).

Overall, for successful breeding, the characteristics of the flat roofs seem to be less important than those of the surrounding landscape^34,47^. Territories of successfully breeding Oystercatcher pairs in the study area were surrounded by a larger proportion of industrial areas. Industrial areas in the study area provided a high availability of flat gravel roofs as potential nesting sites^71^. Furthermore, they were usually also characterised by a large proportion of urban lawns, whose importance for foraging is highlighted below^55^. The availability of foraging habitats with abundant and easily accessible prey during the breeding season is known to be another important driver of Oystercatcher breeding performance^31,41,46^. This is especially true for urban areas where potential foraging habitats are generally scarce. Dijkstra and Dillerop^47^ observed that Oystercatchers on urban rooftops usually breed in proximity to their foraging habitats. In our study, territories of successful breeding pairs had a larger proportion of urban lawns and were closer to sports pitches than the random sites, both of which are used for foraging. Furthermore, the GLM analyses revealed that the distance to the nearest sports pitch was the most important factor for successful breeding in the study area. This can be explained by the outstanding importance of sports pitches as foraging sites for the species. Low-cut turfgrass systems such as sports pitches can be very rich in earthworms^72^, the main food source for inland breeding Oystercatchers^43,45,47^. The high abundance of earthworms is especially promoted by intensive management of the pitches. In particular, the frequent mowing with mulching mowers, which leave fine turfgrass clippings on the pitches, favour the food supply for earthworms^72^. The short turfs and soft soils of sports pitches also make prey easily accessible for probing Oystercatchers.

Although urban lawns and short-turf grasslands can have a similar structure and were widely available in the study area, they only played a minor role in Oystercatcher foraging. This was mainly due to differences in soil moisture, which affects the availability and accessibility of earthworms in the upper soil layer^62,73^. While decreasing NDVI values in urban lawns and agricultural grasslands indicate an increasing impact of drought over the rearing period, sports pitches were regularly irrigated and thus steadily characterised by a higher NDVI (i.e. higher soil moisture). As a result, they provided a high availability and accessibility of prey throughout the whole breeding season. This may be particularly important during periods of droughts, which have become more frequent in recent years (including the study period) due to climate change and can lead to food shortage for waders^62,69,74^. It is well known that the risk of breeding loss increases when adults have to expend more effort on foraging and leave their chicks alone for a longer time (Ref.^47^, cf.^37^). The fact that nesting sites of Oystercatcher pairs without nesting success were further away from sports pitches emphasises that this foraging habitat is of vital importance for the breeding performance of Oystercatchers in urban areas.

### Limitations of the study

Due to the low number of Oystercatcher territories in the study area (N = 24), larger-scale studies in different geographic locations and over multiple years are needed to deepen the results of our study. Although other authors also reported high number of Oystercatchers breeding on urban rooftops throughout Central and Western Europe (e.g.^33,34,47^), information on breeding success of roof-nesting Oystercatchers is still scarce. Therefore, future studies on breeding performance of Oystercatchers nesting on flat roofs would be very useful to draw more general conclusions for conservation. Our study does not provide direct evidence for the drivers of nest and chick survival. Therefore, detailed nest monitoring is required to shed more light on the causes of breeding losses of roof-nesting Oystercatchers compared with ground nesters in more natural habitats. Since it has recently been found that a lower level of human disturbance during the COVID-19 lockdown has affected the occurrence of ground-nesting shorebirds^11,12^, it could be questioned to what extent reduced anthropogenic activity has affected the results of our study in an urban environment. However, as people generally have no access to the roofs used for breeding at any time (i.e. also before and after the lockdown), we assume that the effects of COVID-19-related regulations on the results of our study are negligible. This is underlined by our own observations suggesting that some rooftops in the study area have been used for breeding for at least 20 years. In addition, we frequently observed Oystercatchers foraging on sports pitches only a few metres next to people doing sports during the main rearing period in 2020 (i.e. after the strict COVID-19 regulations have been cancelled at the beginning of May 2020). To dispel any doubts, we nevertheless recommend further studies covering different breeding periods, especially because they also could provide important information on how breeding success and foraging habitat use might vary between different geographic locations and years with different weather conditions.

## Conclusions and implications for conservation

Our study has shown that urban gravel rooftops can provide well-suited breeding conditions for the Oystercatcher. Although urban roof-nesting Oystercatchers cannot compensate for the large-scale decline of the species in its coastal ranges, they could at least help to maintain viable populations of the species in the long term. This is mainly due to the protection from mammalian predators, which leads to higher reproductive success of the species compared to coastal and agricultural habitats. As gravel and green roofs can also be suitable for nesting of other ground-nesting birds and have other ecological benefits (e.g.^37–39,75^), they should be favoured wherever possible when constructing flat roofs in urban areas. However, the findings of our study also suggest that the surrounding landscape is another important factor for the Oystercatcher^34,47^. In particular, successful breeding of urban Oystercatchers depends on habitats that provide a large amount of prey that is easily accessible throughout the breeding season. In our study, this was especially true for sports pitches, which provided a high availability of earthworms due to their moist and soft soils. Other irrigated lawns, such as those in urban parks or on golf courses can provide similar conditions, but were scarce in the study area. As long as the soil is moist enough, short-grass pastures, lawns or road verges can also be suitable for foraging. However, they were of minor importance our study due to drought.

Despite the advantages of breeding on urban rooftops, the species’ strong territory fidelity makes it highly sensitive to the rapid environmental changes in urban areas. For instance, increasing drought due to climate change as well as the conversion from natural grass pitches to artificial turf can severely reduce the availability of foraging sites for Oystercatchers in cities. In addition, the use of pesticides to control earthworms on sports turf can severely reduce the abundance of prey^62,72^. These examples highlight that the value of urban ecosystems for bird conservation should be better integrated into urban planning and habitat management^17^. A large proportion of urban green space is particularly beneficial for bird conservation in urban areas^5^. This also applies to low-cut habitats such as sports pitches, golf courses, lawns as well as short-grass pastures and wastelands, which are important for ground-foraging species such as the Oystercatcher^41,47,76^.

The increasing shift of the species from its original breeding habitats to urban rooftops also highlights the severe challenges for Oystercatcher conservation in rural landscapes. Conservation measures in the wider countryside must therefore include both large-scale actions to improve the quality of breeding habitats for Oystercatchers and predator control that may also help to mitigate the declines in other shorebirds besides the Oystercatcher^30,31,61,77^.

## Data Availability

The data that support the findings of this study are available from the authors upon reasonable request.
